# Pathophysiology of Atherosclerosis

**DOI:** 10.3390/ijms23063346

**Published:** 2022-03-20

**Authors:** Shifa Jebari-Benslaiman, Unai Galicia-García, Asier Larrea-Sebal, Javier Rekondo Olaetxea, Iraide Alloza, Koen Vandenbroeck, Asier Benito-Vicente, César Martín

**Affiliations:** 1 Department of Biochemistry and Molecular Biology, Universidad del País Vasco UPV/EHU, 48940 Leioa, Bizkaia, Spain; sjebari001@ikasle.ehu.eus (S.J.-B.); iraide.alloza@ehu.eus (I.A.); koen.vandenbroeck@ehu.eus (K.V.); 2 Biofisika Institute (UPV/EHU, CSIC), Barrio Sarriena s/n., 48940 Leioa, Bizkaia, Spain; u.galiciag@gmail.com (U.G.-G.); alarrea040@ikasle.ehu.eus (A.L.-S.); 3 Fundación Biofisika Bizkaia, Barrio Sarriena s/n., 48940 Leioa, Bizkaia, Spain; 4 Cardiology Department, Basurto University Hospital, 48013 Bilbao, Bizkaia, Spain; javiergregorio.rekondoolaetxea@osakidetza.eus; 5 Inflammation & Biomarkers Group, Biocruces Bizkaia Health Research Institute, 48903 Barakaldo, Bizkaia, Spain; 6 Ikerbasque, Basque Foundation for Science, 48013 Bilbao, Bizkaia, Spain

**Keywords:** atherosclerosis, endothelial dysfunction, atheroma plaque, risk factors, microbiota, ocLDL, microRNA, lncRNA

## Abstract

Atherosclerosis is the main risk factor for cardiovascular disease (CVD), which is the leading cause of mortality worldwide. Atherosclerosis is initiated by endothelium activation and, followed by a cascade of events (accumulation of lipids, fibrous elements, and calcification), triggers the vessel narrowing and activation of inflammatory pathways. The resultant atheroma plaque, along with these processes, results in cardiovascular complications. This review focuses on the different stages of atherosclerosis development, ranging from endothelial dysfunction to plaque rupture. In addition, the post-transcriptional regulation and modulation of atheroma plaque by microRNAs and lncRNAs, the role of microbiota, and the importance of sex as a crucial risk factor in atherosclerosis are covered here in order to provide a global view of the disease.

## 1. Introduction

Atherosclerosis is a disease that is characterized by the accumulation of lipids, fibrous elements, and calcification within the large arteries. This process is initiated by endothelium activation, followed by a cascade of events, which implies the vessel narrowing and activation of inflammatory pathways leading to atheroma plaque formation. Altogether, these processes result in cardiovascular complications that remain as the leading cause of death worldwide.

In this study, we aimed to review the mechanism of development of atherosclerosis, including endothelial dysfunction, fatty streak formation, fibrous plaque formation, and plaque rupture. This review approaches the pathophysiology of atherosclerosis from a broad perspective, examining the pathological and biochemical processes of atherosclerotic plaque formation and growth. In addition, this review summarizes the current understanding of the involvement of microRNAs and lncRNAs in post-transcriptional regulation and modulation of atheroma plaque and atherogenesis. Non-traditional drivers of atherosclerosis—such as disturbed sleep, physical inactivity, the microbiome, the role of microbiota, and the importance of sex as a crucial risk factor in atherosclerosis are covered here in order to provide a global view of the disease.

## 2. Endothelium

The vascular endothelium is a heterogeneous monolayer formed by endothelial cells (ECs), which face the luminal side of all blood vessels, representing the first barrier for molecules, cells, or pathogens circulating in the bloodstream [1]. In large vessels, the vessel wall is lined with a single EC layer called the endothelium, which, together with collagen and elastic fibers, forms the luminal layer of the vessels or the intima. ECs are in intimate contact with tunica media consisting of vascular smooth muscle cells (VSMC) and elastic and collagenous tissue. Finally, surrounding this layer is tunica adventitia, which is composed mainly of a dense matrix of connective tissue. On the other hand, the walls of arterioles and venules are composed of the same three layers as the larger vessels, although the media and adventitia are much thinner and less pronounced. Finally, post-capillary venules completely lack media and adventitia and only consist of ECs and a basement membrane [2].

Endothelium is strategically located between circulating blood and tissues, working as a sensor and transducer of signals by the production of biologically active substances. All changes in circulating blood are perceived by the endothelium, which then mediates signal transduction to the other layers of the vascular wall. Such changes include mechanical stress (elongation and wall shear stress (WSS)), as well as changes in the concentration of metabolic factors.

Different mechanical forces acting on the arterial wall modulate several physiological functions, such as the regulation of homeostasis, vascular tone, and vascular integrity. Apart from their role in homeostasis, the involvement of hemodynamics in vascular disease development has been shown to be crucial in the pathology of atherosclerosis, affecting both disease onset and progression. The main forces acting on the arterial wall include both tensile stress induced by blood pressure and WSS, a tangential force to the vessel wall induced by blood flow that plays an important role in atherogenic hemodynamics [3].

Vessel segments with low WSS or highly oscillatory WSS appear to be at the highest risk for development of atherosclerosis [4,5]. WSS change can directly affect the morphology and function of the vascular endothelium and stimulate the migration and proliferation of VSMCs and mononuclear cells [6]. Whether low or unstable, changing WSS is an indicator to evaluate hemodynamic changes that are closely related to atherosclerosis [7,8].

The magnitude and direction of WSS are recognized by mechanosensors on the endothelium and are transduced as biochemical signaling. WSS-induced mechanotransduction regulates the expression of numerous genes involved in cell morphology, adhesion, and proliferation.

**Figure 1:**
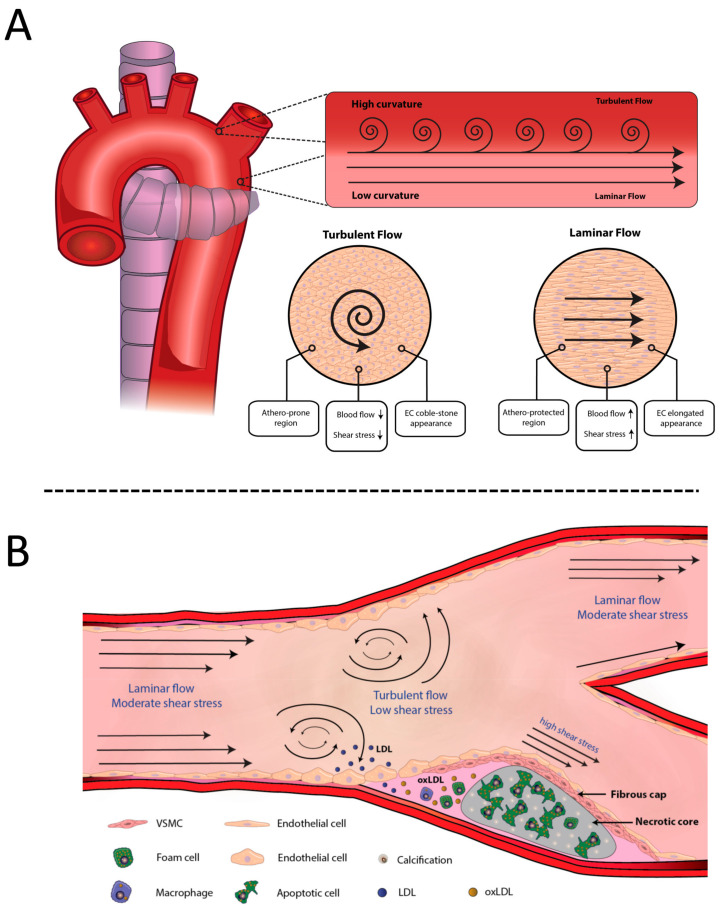
Effect of flow and WSS patterns at arterial bifurcations on atherosclerotic plaque development. (**A**) In straight vessel segments, physiological WSS with laminar flow leads to ECs and shows a quiescent characteristic flattened shape when flow disturbance occurs. Lower WSS at the outer vessel wall causes ECs to adopt a cobblestone appearance. (**B**) Turbulent flow occurs at bifurcations and branch points where the arterial curvature is higher due to flow separation. Disturbed laminar flow or turbulent flow reduces WSS and promotes endothelial dysfunction and LDL infiltration, which constitutes the first step of atheroma plaque formation. On the contrary, low curvature areas of the vascular system subjected to higher shear stress are athero-protected.

For example, shear stress induces characteristic EC alignment [9,10] (Figure 1A). In tubular or straight regions of arteries, where the WSS presents a laminar flow, ECs show a flattened shape and an elongated alignment in the direction of the flow [11]. However, at the bifurcation or high vessel curvature sites, flow disturbance occurs and, as a consequence of the turbulent and reversal flow with lowered WSS at the outer vessel wall, ECs augment their volume by adopting a cobblestone appearance [12] (Figure 1A). Moreover, hemodynamic forces determine the early development of localized atherosclerotic plaques that are not randomly distributed, neither in experimental animal models nor in humans [13,14]. Atherosclerotic lesions mainly occur in regions characterized by low WSS and flow separation (Figure 1B) and most frequently involve branch points and bifurcations. The extensive correlative data indicating that low shear or disrupted flow accounts for the localization of atherosclerosis lesions emphasize the importance of arterial branches and bifurcations when performing a diagnosis of atherosclerotic lesion development or progression.

Endothelium modulates the tone of underlying vascular smooth muscle; maintains a non-adhesive luminal surface; and mediates hemostasis, cellular proliferation, and inflammatory and immune response in the vascular wall [15]. In fact, endothelium releases both agonists and antagonists in order to balance the effect in both directions. For instance, ECs are able to produce both coagulants or anti-coagulants, vasodilators or vasoconstrictors and pro-inflammatory or anti-inflammatory molecules [15,16].

## 3. Atherosclerosis Initiation and Fatty Streak Formation

Atherosclerosis initiates upon endothelial dysfunction accompanied by low-density lipoprotein (LDL) retention and its modification in the intima [17,18]. Modified LDLs, together with additional atherogenic factors, promote the activation of ECs, leading to monocyte recruitment within the intima. Modified LDLs are avidly captured by differentiated monocytes and VSMC, which promote foam cell formation [19,20]. In addition, several inflammatory signaling pathways are activated, allowing the fatty streak formation, which represents the first sign of atherosclerosis and is characterized by a substantial accumulation of lipids both within the cells (macrophages and VSMC) and the extracellular media [21].

### 3.1. Endothelial Dysfunction in Atherosclerosis Development

Disruption of the mechanisms involved in vascular homeostasis regulation leads to endothelial dysfunction [9,12,22]. Briefly, when ECs lose their ability to maintain homeostasis, vessel walls are predisposed to vasoconstriction, lipid infiltration, leukocyte adhesion, platelet activation, and oxidative stress, among other things [23,24]. Together, these induce an inflammatory response that is considered the first step of atheromatous plaque formation: the fatty streak [12,22]. In addition, endothelial dysfunction also plays a remarkable role in subsequent steps of atherosclerosis by participating in plaque development and in its rupture in the last steps of atherosclerosis [22]. Therefore, an increased endothelial dysfunction is considered an early indicator of atherogenesis [25,26].

#### 3.1.1. Hemodynamic Forces and Endothelial Dysfunction

Hemodynamic forces constitute a local risk factor of atherogenesis, as they promote endothelial dysfunction [27]. As indicated above, lesion-prone regions are mainly located in areas where the laminar flow is disturbed due to flow separation, recircularization, or reattachment [28]. This turbulent flow creates temporal and spatial gradients, which result in a higher oscillatory index and a lower shear stress [11,29]. In addition, a disturbed flow also favors lipoprotein infiltration into the vessel intima, firstly because LDLs remain in those areas for longer periods of time, and secondly because turbulent flow induces a physical disruption of endothelial integrity [30,31], thus facilitating lipoprotein infiltration [30,32,33]. In addition, another fundamental link between hemodynamic forces and atherogenesis relies on the expression of diverse endothelial genes regulated by blood mechanical stimulus [34,35,36].

The effect of shear stress over endothelial gene expression has been studied during the last 20 years; more than 40 genes implicated in the process have been discovered so far [37,38,39,40,41,42]. Among them, several atherogenic genes, such as monocyte chemoattractant protein 1 (MCP-1), which induces monocyte permeation into the arterial wall [43,44,45], and platelet-derived growth factors (PDGFs), which enhance VSMC migration [46,47,48], are upregulated in ECs. Interestingly, research evidence revealed shear stress response elements (SSREs) in the promoters of these genes and others, such as eNOS or platelet adhesion molecule-1 (PECAM-1), which contribute to plaque development [49,50,51,52]. Moreover, the combination of two or more SSREs in the same promoter could have a synergistic effect that enhances the expression of those genes [53]. On the other hand, in straight regions of the vasculature where the laminar flow drives high shear stress in the endothelium, some pro-atherogenic genes are downregulated, while genes that induce cell-cycle growth arrest or increase the antioxidant capacity are upregulated. Indeed, a long exposure of ECs to undisturbed laminar flow promotes the upregulation of endothelial nitric oxide synthases (eNOSs), thus increasing their nitric oxide (NO) synthesis capacity [13,49].

These findings suggest a differential molecular response in endothelium depending on the blood flow pattern, highlighting the role of hemodynamic forces in endothelial dysfunction.

#### 3.1.2. The Role of Nitric Oxide in Endothelial Dysfunction

**Figure 2:**
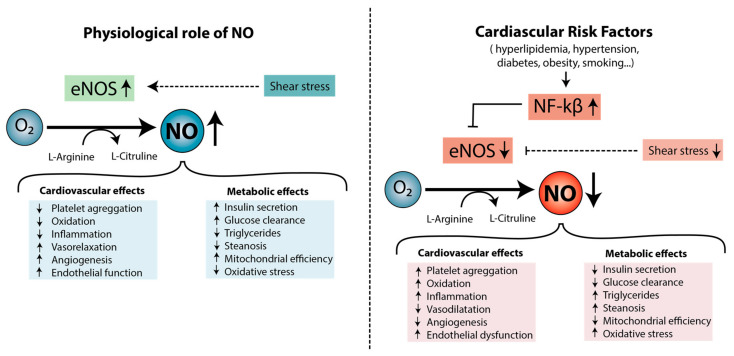
Nitric oxygen regulates cardiovascular metabolism and is compromised in the presence of cardiovascular risk factors. eNOS catalyzes the production of NO from L-arginine. NO is an essential metabolite that inhibits the progression of atherosclerosis improving vasorelaxation, angiogenesis, endothelial function, insulin secretion, glucose clearance, and mitochondrial efficiency. On the other hand, it reduces oxidative stress, inflammation, plasma lipid levels, and stenosis. Cardiovascular risk factors, such as hyperlipidemia, hypertension, and diabetes, inhibit eNOS activity upon NF-kβ induction, reducing NO and promoting atherosclerosis development.

Endothelial dysfunction is also explained through a reduction in NO bioavailability [54]. NO is synthesized from L-arginine in ECs in a reaction catalyzed by eNOS and diffuses across cell membranes, reaching the smooth muscle tissue of the artery wall. NO promotes smooth muscle fiber relaxation, known as endothelium-dependent vasodilatation [55,56], and is considered an athero-protective molecule, because it counteracts atherogenesis and its complications. Specifically, NO is involved in the reduction of platelet aggregation; tissue oxidation and inflammation; the activation of thrombogenic factors; and cell growth, proliferation, and migration, among other things [57,58,59,60] (Figure 2). Moreover, it maintains metabolic homeostasis, as it reduces triglyceride content and steatosis and increases insulin synthesis, glucose clearance, and mitochondrial efficiency [61]. However, in the presence of cardiovascular risk factors, such as hyperlipidemia, hypertension, smoking, or diabetes, NO production is reduced as a consequence of the increased oxidative stress, which is commonly associated with those pathologies [28,62,63]. Oxidative stress promotes the synthesis of pro-atherogenic cytokines (TNF-α and interleukins IL-1 and IL-6), adhesion molecules (VCAM-I and ICAM-I), and chemokines (MCP-1) through NF-kB activation mediated by heat-shock proteins (HSP-60). These mediators inhibit the activity of eNOS and, consequently, NO production [12] (Figure 2). In fact, studies carried out in hypercholesterolemic patients demonstrated an impaired endothelium-dependent vasodilatation due to a defect in the bioavailability of NO [64]. Hypertensive patients also show a defect in the endothelium-derived NO system that may explain the abnormal endothelium-dependent vasodilatation [65,66].

### 3.2. LDL Infiltration

Accumulation of LDL in plasma favors transendothelial infiltration of circulating LDLs to the intima. Although it has been traditionally accepted that LDLs cross the endothelium by diffusion or paracellularly [67,68,69,70], it is now accepted that transcytosis plays an important role in the transendothelial transport of LDLs [71,72]. More specifically, it has been shown that LDL transcytosis is mediated by scavenger receptor B1 (SR-B1) and activin A receptor-like type 1 (ALK1) receptor of the endothelium, which differs from the classical LDL endocytosis pathway mediated by LDLR [73]. SR-B1 and ALK1 receptors co-localize with *caveolae*, indicating that LDL transcytosis by SRB1 and ALK1 is mediated by a *caveolae*-dependent mechanism [74,75]. Moreover, an absence of caveolin-1, the major structural protein of *caveolae* in ECs [76], significantly impairs LDL transport and retention within the arterial wall [77,78], and increased levels of caveolin-1 have been found in atherosclerotic lesions [79]. Although additional experiments are needed to elucidate the specific transport mechanism of both receptors, these findings suggest that *caveolae*-dependent LDL uptake plays an important role in LDL transcytosis [80,81].

Although this review only summarizes the role of ECs in LDL infiltration, it is important to note that other factors, such as the glycocalyx [82], pericytes [83], the subendothelial extracellular matrix [84,85], and the role of shear stress [32], should be considered.

#### LDL Modifications in the Intima

Once in the subendothelial space, trapped LDL particles are oxidized, a process facilitated by the absence of protective plasma antioxidants, such as tocopherol, ascorbate, urate, apolipoproteins, or serum albumin [86,87]. Oxidized LDLs are key inflammatory components that promote atherosclerotic plaque development, as they contain oxidized lipids and products derived from their degradation that contribute to the physiopathology of the disease [88,89].

LDLs can be oxidized by free radicals present in the extracellular media, such as superoxide (O_2_^•–^), hydroxyl radicals (^•^OH) [90], and others, such as HClO, produced by the surrounding cells [91,92]. Additionally, LDLs can be directly oxidized by the enzymatic activity of phospholipases and lipoxygenases [93,94]. In fact, the lipoxygenase pathway has been highlighted to explain the initiation of LDL oxidation [95]. Interestingly, LDL receptor-related protein (LRP) is involved in LDL recruitment, and 12/15 lipoxygenase is translocated to the membrane where oxidation of LDL cholesterol esters takes place [94].

Independent of the mechanism involved in the initiation of the LDL oxidation, the process is characterized by the loss of the antioxidants carried by LDLs, including alpha-tocopherol and carotenoids [96,97]. This is followed by the small degradation of polyunsaturated fatty acids (PUFAs), mostly arachidonic and linoleic acids, which are oxidized to hydroperoxides. The latter leads to the formation of conjugated dienes and, upon further oxidation, short-chain aldehydes [98].

On the other hand, apoB-100, the major protein of the LDL, also suffers different modifications as a consequence of the oxidative environment. For instance, aldehydes generated from lipid oxidation form adducts with lysine residues of apoB-100. Instead, apoB-100 can be directly modified predominantly at tyrosine residues by oxidizing agents [99]. These modifications inhibit LDL-LDLR recognition, thus increasing the uptake of LDL particles through non-regulated receptors [87].

Depending on the LDL oxidation level, oxidized LDLs are classified as minimally modified LDL (mmLDL) or extensively oxidized LDL (oxLDL) [100]. Mm-LDLs differ chemically from unmodified LDLs but are still recognizable by the LDLR and therefore are internalized through regulated pathways. However, modified lipids within these particles act as bioactive molecules conferring other biological activities not shown in unmodified LDLs [100,101]. These bioactive lipids might induce a pro-inflammatory response in ECs and macrophages [102,103,104]. On the other hand, when LDLs are extensively modified, they become unrecognizable by the LDLR, while allowing recognition by a range of scavenger receptors [105,106,107,108]. Oxidative modifications of apoB-100 underlie this lack of affinity toward LDLR and the increased affinity for the scavenger receptors. Moreover, oxLDLs are able to escape from proteoglycan retention, hence favoring their non-regulated uptake by *scavenger* receptors [84,109]. Once internalized, products derived from oxLDL trigger the expression of inflammatory molecules in macrophages, as outlined later in this review.

It is important to note that, even with LDL oxidation being the most common modification, a range of LDL modifications contributing to atherosclerosis development have been well described in other works, including glycosylation, acetylation, and aggregation [107,110,111,112,113].

### 3.3. Endothelial Activation

Endothelial stimulation, also known as endothelial type I activation, occurs when inflammatory agents induce a response such as a change in microvascular tone, permeability, or leukocyte diapedesis [114,115]. This phenomenon is an acute response with short-term functional and morphological changes and does not require de novo protein synthesis or gene upregulation [115]. However, in response to certain proinflammatory agents, such as IL-1, TNF, endotoxins, modified lipoproteins, and advanced glycosylation end products (AGE), as well as disturbed flow derived biomechanical stimulation, the endothelium can undergo a sustained phenotypic modulation, known as endothelium type II activation [114,115]. This activation leads to a complex inflammatory response that starts with an increased NF-kB production within the ECs, in response to the aforementioned stimulus. NF-kB upregulates the expression of leucocyte adhesion molecules, such as VCAM-1 and ICAM-1; secreted chemokines, such as MCP-1 and IL-8 [116,117]; and prothrombotic mediators, such as plasminogen activator inhibitor or tissue factor.

### 3.4. Monocyte Recruitment and Foam Cell Formation

Activated ECs induce selective monocyte recruitment into the intima. This process has been visualized in vitro [118], and it can be summarized in the rolling, adhesion, activation, and transmigration of monocytes, as has been well documented [119,120,121].

Briefly, monocyte recruitment starts with monocyte capture and rolling over the endothelium, which is mainly mediated by P-selectin [122,123]. Monocyte-rolling is then reduced, and monocytes remain firmly attached to the endothelium [124], a process mediated by the binding of monocytes integrins to VCAM-I and ICAM-I of ECs [123,125,126]. In addition, while rolling over the endothelium, monocytes are activated by endothelial surface-bound chemokines [127], such as CXCL1, CXCL2, CXCL4, and CCL5, and this increases monocyte adhesiveness [124]. Afterward, monocytes transmigrate into the intima space. This movement comprises the crossing throughout the EC barrier, its basement membrane, and the pericyte layer [123,128]. The migration process is held by chemokines, which have been previously secreted in response to proinflammatory signals.

Regarding monocyte recruitment, MCP-1 (also named as CCL2) is the most frequent chemokine mediating monocyte transmigration; however, the effect of other chemokines, such as CCL3, CCL4, and CCL5, has also been studied [129,130]. MCP-1 is produced mainly by ECs, smooth muscle cells, and monocytes and macrophages of the intima, and its expression is upregulated after proinflammatory stimulus or tissue injury, favoring the transendothelial migration of circulating monocytes from the plasma to the intima [131]. This process is mediated by the paracellular and transcellular routes [120,123,132]. In the paracellular route, monocyte migration is favored through EC junctions, due to the redistribution of junctional molecules in the inflamed endothelium [133,134]. In addition, some endothelial junction molecules actively mediate this type of migration [135,136]. On the other hand, in the transcellular route, cells migrate through the body of ECs; however, this type of transmigration has been observed in only 10–30% of the events in vitro [137,138]. New advances in live cell imaging will clarify this field [118,139]. Finally, monocytes cross the EC basement membrane, which is composed of a network of laminin and collagen, and the pericyte sheath, which is found in most venules [132,140].

Once in the intima, monocytes are differentiated into macrophages that can be polarized to the M1 (pro-inflammatory) or M2 (anti-inflammatory) phenotype [141,142]. Nonetheless, macrophages show sensitivity to the changes in inflammatory environment, and, in response to new signals, they are able to switch their phenotype from pro-inflammatory to anti-inflammatory [143,144,145]. Macrophage plasticity is fundamental for a successful response with M1 predominating in disease progression and M2 in regression [142,146]. M1 macrophages release inflammatory cytokines and chemokines and produce NO and reactive oxygen species (ROS), which promote monocyte recruitment and inflammatory response propagation [144]. In addition, macrophages express a battery of receptors that mediate the internalization of modified and non-modified LDLs. As previously mentioned, retained lipoproteins in the intima are prone to suffer modifications due to the inflammatory environment, allowing their internalization through CD36, SRA-l, and LOX-I scavenger receptors [105,106,107]. It is important to underline that the expression of those receptors is not downregulated by cholesterol uptake. Thus, in an atherosclerotic context, where oxLDL content is significantly enhanced, cells internalize higher amounts of oxLDLs. Within the cells, oxLDLs are degraded in the lysosomes, and the lipoprotein-contained cholesterol is esterified by acyl CoA:cholesterol acyltransferase (ACAT) in the endoplasmic reticulum (ER). Cholesterol esters are stored as lipid droplets located both in the cytoplasm or linked to the ER [147,148]. Hydrolysis of these packed cholesterol esters mediated by neutral cholesterol ester hydrolases, such as nCEH and NCEH1, generates free cholesterol that is transferred from macrophages to apoA1 or HDLs (high-density lipoprotein), an important step for the removal of cholesterol excess from peripheral tissues [149]. This process is mediated by ABCA1 and ABCG1 ATP-binding cassettes and SR-B1, cholesterol transporters that play an important role mediating cholesterol efflux from the cells and preventing foam cell formation [105]. However, the pro-inflammatory microenvironment of atherosclerotic lesions impairs the ABCA1 efflux system, both in M1 and M2 macrophages, and promotes foam cell accumulation, as shown in experiments with murine macrophages contributing to plaque development [150,151].

In addition, the excess of lipid uptake by macrophages perpetuates the inflammatory response, and oxLDLs induce signaling cascades that activate NF-kB targets [152,153,154], which maintain EC activation, monocyte recruitment, and foam cell formation [146]. The uptake of oxLDLs by macrophages could be considered a protective mechanism, as they remove cytotoxic elements from the intima. However, the increased migration of monocyte to the intima and the subsequent differentiation into macrophages lead to a large number of foam cells inducing the growth of the atherosclerotic lesion [147]. Therefore, cholesterol accumulation is considered a hallmark of atherosclerotic lesions [155,156].

An accumulation of cholesterol in the subendothelial compartment also promotes the formation of cholesterol crystals both inside and outside the cells and contributes to the development of atherosclerotic plaques [157,158,159]. This process has been monitored both outside and inside the cells, in macrophages incubated with oxLDLs [160]. Although cholesterol crystals are a common feature of advanced atherosclerotic lesions, they are present also in early plaques and can be used as a marker of early atherosclerosis development [161]. Cholesterol crystals within the plaque activate NLRP3 inflammasome in macrophages, leading to activation of pro-inflammatory pathways. Inflammasomes are cytosolic multiprotein complexes of the innate immune system responsible for the activation of inflammatory pathways [162]. Although NLRP3 activation and assembly is not fully understood, it is known that its activation leads to caspase-1 activation. Caspase-1 subsequently cleaves the proinflammatory IL-1 family of cytokines into their bioactive forms, IL-1β and IL-18, contributing to inflammation [88]. It has been suggested that uptake of oxLDLs mediated by the CD36 receptor is responsible for NLRP3 activation [88]. Apparently, the CD36 scavenger receptor, along with TLR4-TLR6, takes up oxLDL, which results in intracellular cholesterol crystals. These crystals cause lysosomal destabilization [163], inducing the release of lysosomal contents, such as cathepsins or reactive oxygen species [164], leading to NLRP3 inflammasome assembly and the subsequent activation of caspase-1.

### 3.5. Contribution of VSMCs to Foam Cell Population

VSMCs located in the intima are also able to internalize oxLDL in a non-regulated way through different scavenger receptors, such as SR-A, CD36, and LOX-1 [165,166,167,168,169]. Indeed, their contribution to the sum of foam cell population within the plaque is significant [20]. In addition, VSMCs of the intima express fewer ABCA1 transporters than the ones of the tunica media [170]. Therefore, the balance between cholesterol input and output is tilted in favor of cholesterol accumulation and foam cell formation. At least 50% of the foam cells in the human coronary intima are VSMC-derived rather than monocyte-derived, underlying the importance of VSMCs in atherosclerosis development [170].

## 4. Fibrous Plaque Development

**Figure 3:**
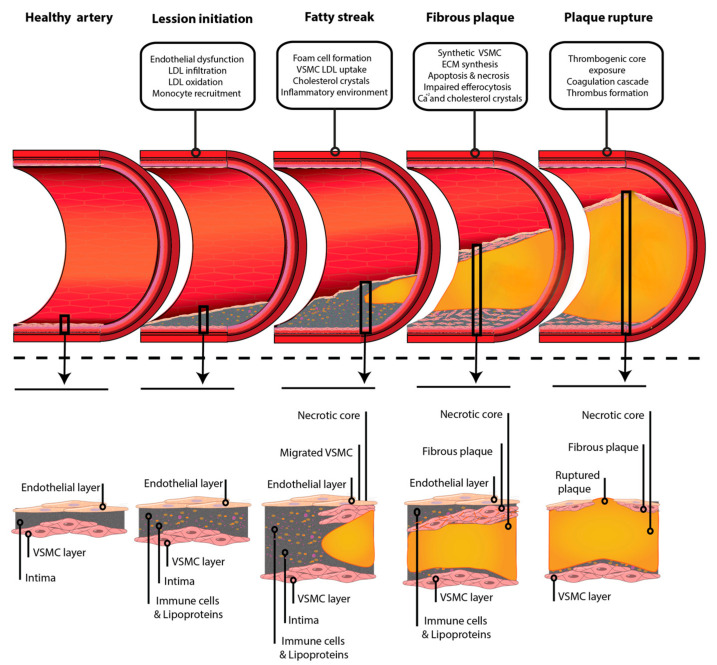
Schematic representation of atheroma plaque formation from a healthy artery to plaque rupture underlying the most important events that contribute to its development in each stage.

During fibrous plaque development, atheroma plaques undergo a transition from the fatty streak to intimal growing, a step characterized by the presence of a cell-free and lipid-rich area known as the necrotic core (Figure 3). To stabilize the plaque, the necrotic core is covered by fibers, thus developing a fibrous cap. The necrotic core and the fibrous cap constitute the hallmark of advanced atherosclerosis [171], and atheroma plaque regression is unlikely to happen in this stage [172,173].

### 4.1. Fibrous Cap

The fibrous cap is a subendothelial barrier between the lumen of the vessel and the atherosclerotic necrotic core consisting of VSMCs that have migrated to the luminal side of the artery and extracellular matrix (ECM) derived from VSMCs [174,175]. The role of the fibrous cap is to serve as a structural support to avoid the exposure of prothrombotic material of the core that otherwise would trigger thrombosis [174].

At the physiological situation, differentiated VSMCs of the tunica media show a contractile phenotype that regulates the blood vessel diameter and blood flow [176,177,178]. However, in response to injury, VSMCs switch their phenotype to the synthetic one in which migratory and proliferation activities prevail [179,180]. For that purpose, neighboring cells activate the healing process by producing several growth factors, which include epidermal growth factor, fibroblast growth factor, insulin-like growth factor, platelet-derived growth factor (PDGF), transforming growth factor-β (TGF-β), and vascular endothelial growth factor (VEGF) [179]. In atherosclerosis, in response to the growth factors produced by foam cells (VSMC- or macrophage-derived) or ECs of the intima, VSMCs from the tunica media migrate to the intima [175,179,181,182,183]. Moreover, IL-1 produced by macrophages enhances the endogenous production of PDGF by VSMC, and, once in the intima, it autocrinically leads to their proliferation [184,185]. In addition to migration and subsequent proliferation, VSMCs with a synthetic phenotype also increase the production of ECM components, such as interstitial collagen, elastin, and proteoglycans [183,186,187,188]. These proliferating VSMCs, along with ECM production, generate a fibrous cap that will cover the developing atherosclerotic plaque, thus surrounding the lesion and preventing its rupture [189]. Interestingly, if the mitogen production does not cease, VSMCs do not switch back to the contractile phenotype [176,177,190,191], which facilitates lesion development. Fibrous-cap features, such as thickness, cellularity, matrix composition, and collagen content, are important determinants of plaque stability [192,193,194].

### 4.2. Necrotic Core

The necrotic core constitutes the nucleus of the atherosclerotic plaques. Covered by the fibrous cap, the necrotic core consists of a lipid-laden hipocellular region with reduced supporting collagen [195,196,197]. While atherosclerotic plaque develops, the necrotic core increases its size mainly as a consequence of two factors, macrophage death and impaired efferocytosis, a process that removes apoptotic cells. Both events contribute to an inflammatory microenvironment, oxidative stress, and thrombogenicity and promote the death of neighboring cells, such as VSMCs, increasing plaque vulnerability [198].

In the early stages of atherosclerosis, macrophage apoptosis is programmed through the coordinated caspase system, leading to cell death and efferocytosis [199]. However, when atherosclerosis develops, apoptosis enhances in the macrophage and VSMCs due to increased oxidative stress, the activation of receptors involved in death signaling, the inhibition of survival pathways, and nutrient deprivation [200]. At this step, the phagocytic activity of the macrophages is not able to handle the accumulation of apoptotic cells, which undergo a secondary necrotic death with the concomitant release of intracellular oxidative and inflammatory components. This situation aggravates inflammation and enhances the death of neighboring cells [201]. Efferocytosis also becomes defective in advanced atherosclerosis because the activity of several receptors that mediate the process, such as MerTK, LRP1, CD36, and SR-B1, is impaired. Moreover, the oxidized phospholipids and oxLDLs present in advanced atherosclerotic plaques inhibit the recognition of apoptotic cells by efferocytotic receptors [201,202,203,204,205]. Efferocytosis impairment in advanced plaques also favors cholesterol crystal accumulation coming from apoptotic cells. At physiological conditions, small cholesterol crystals are rapidly sequestered from interstitial space by phagocytic cells; however, while the lesion advances, phagocytic cells are unable to remove the excess of crystals, which finally increase in size and remain in the subendothelial space [206]. This process activates the complement and increases the proinflammatory state of the plaque. Moreover, as phagocytic cells are unable to internalize large cholesterol crystals by scavenger receptors, their lysosomal content is directly secreted to the interstitial space, promoting a more intense immune response [207,208]. These events promote the death cell accumulation and necrotic core growth. Furthermore, the metalloproteinases released after cell death reduce the size of the fibrous cap and increase plaque vulnerability [209,210]. Finally, apoptotic and necrotic cells liberate tissue factor (TF), which, along with oxidized lipids, increases the thrombogenicity of the necrotic core [211,212].

### 4.3. Plaque Calcification

Atheroma plaque calcification is another hallmark of advanced atherosclerosis. It exists as a bone-like formation within the plaque and is initiated in inflammatory regions with a local decrease in collagen fibers [213,214]. The release of matrix vesicles upon the macrophage and synthetic VSMC death initiates the calcification process of the plaque [215,216,217,218]. The nucleation sites accumulate calcium orthophosphate, which is converted to amorphous calcium phosphate and finally to crystalline structures [213]. In addition, other factors, including reduced levels of mineralization inhibitors or increased osteogenic transdifferentiation, contribute to the calcification process [213]. In particular, pericytes [219] and VSMCs [220,221] transdifferentiate into osteoblast-like phenotypes during atherosclerosis development, acquiring the capacity to generate a mineralized matrix and leading to calcium deposits, as it occurs in bone tissue [222,223,224].

Together, this contributes to microcalcifications, the early stage of the vascular calcification cascade in both intima and media [213,225]. Microcalcifications then evolve into larger calcifications that extend from the bottom of the necrotic core to the surrounding matrix [221]. Although calcification is a hallmark of advanced atherosclerosis (it correlates very well with plaque size), the amount and size of calcium deposits are not correlated with plaque vulnerability, which would rather be linked to other features, such as location, calcification type, or the surrounding environment [213,214,226].

## 5. Plaque Stability and Rupture

### 5.1. Vulnerable Plaque

As mentioned above, atheroma plaques usually develop in branched areas where the WSS is lower. In these areas, low shear stress contributes to local endothelial dysfunction and eccentric plaque build-up. Initially, lumen narrowing is prevented by outward vessel remodeling to maintain a normal lumen and to restore shear stress distribution. However, this prolongs local, unfavorable, low-WSS conditions and aggravates eccentric plaque growth. The eccentric plaques at preserved lumen locations experience increased tensile stress on their shoulders, transforming the lesion into a rupture-prone [227]. A plaque is considered vulnerable when the lesion shows a large necrotic core, a thin fibrous cap, and an increased inflammatory response [228,229] due to the continuous exposure to the pro-atherogenic milieu.

The fibrous cap separates the thrombogenic necrotic core from the circulating coagulation factors and platelets, and its thickness correlates with the vulnerability of the plaque [189]. As a result of VSMC death, ECM production is reduced, and the presence of liberated matrix metalloproteinases (MMP) increases, making the fibrous cap weaker [230] (Figure 3).

As mentioned before, inflammation contributes to plaque development in all of the steps from initiation to plaque rupture. Indeed, in this last stage, its relevance is remarkable, as it promotes the instability of the fibrous cap [231]. Certain pro-inflammatory cytokines, such as IFN-γ, might inhibit collagen production in VSMCs. In addition, inflammatory mediators usually found in atheroma, such as IL-1β, TNF-α, and the CD40 ligand (CD154), may increase MMP expression in VSMCs, as observed in vitro [232,233]. This inflammatory stage is commonly observed in the cap and shoulders of the plaque instead of a generalized inflammation [189,234]. Together, the data show that, when inflammation prevails, the maintenance of the strong and rigid fibrous cap decreases, making the cap unstable and susceptible to rupture when exposed to hemodynamic forces, the most common mechanism of plaque rupture [189,227,231,235].

### 5.2. Plaque Rupture and Thrombus Formation

When the plaque fissures or ruptures, the subendothelial space is exposed to blood, triggering a coagulation process to cover the wound [186,236]. Initially, platelets adhere to the subendothelial collagen and become activated, and more platelets are then recruited and aggregated in the region in order to initiate wound healing [237]. Simultaneously, pro-thrombotic elements of the lipid core are released and come into contact with coagulating factors of plasma. More specifically, the tissue factor of the core reacts with factor VII of the plasma, activating the coagulation cascade that leads to thrombin production, an essential intermediate for fibrin formation [189,238]. Fibrin is an insoluble protein that forms networks of fibrin threads and, along with platelets, covers the lesion, forming a stable and well-arranged structure. This structure is known as the thrombus [231,239].

Although the aim of this process is wound healing, triggering of the biochemical cascade promotes the expansion of the intima to the luminal side. For instance, activated platelets release TGF-β, which, as indicated earlier, promotes the production of interstitial collagen and, therefore, fibrous-cap thickening [240]. Consequently, the atherosclerotic lesion expands, leading to lumen constriction. This all supposes an absence of clinical complication.

### 5.3. Clinical Complications

Thrombus development triggers a range of reactions that makes the lesion more fibrous and stable and, therefore, less prone to rupture. However, due to plaque growth, the risk of vessel obstruction increases. Consequently, blood flow in coronary arteries is reduced, generating ischemic cardiopathies, such as cardiac insufficiency or angina pectoris [231,241,242]. Moreover, if the obstruction is complete or almost complete, it leads to myocardial infarction or stroke [243]. Detachment of the thrombus from the arterial wall would produce a clot, known as the embolus, that circulates within the cardiovascular system. Eventually, the embolus lodges in distal arteries, where it obstructs blood flow, leading to local ischemia, organ dysfunction, or potential infarction [244].

However, if the inflammatory response ceases in time, for example, due to a lipid lowering treatment, a stable plaque with enough lumen for correct blood circulation would be acquired [231].

## 6. Inflammation in Atherosclerosis

As indicated through the previous sections of this review, several inflammation processes participate in all phases of atherosclerosis [245,246] At early stages of atherosclerosis, LDLs accumulate in the subendothelial region, where they become modified. VSMCs exposed to modified LDL release chemoattractants, including chemokine 2 (CCL2) and CCL5 [247,248], which promote the recruitment of monocytes [249]. Moreover, ox-LDL and mm-LDL are able to induce a pro-inflammatory response in ECs and macrophages, increase endothelial damage, and recruit leukocytes. Furthermore, mmLDLs can bind to the TLR2 and Class 4 of PRRs and induce the secretion of pro-inflammatory cytokines IL-1β, IL-6, and TNF-α in an NF-kB-dependent fashion [250,251]. CD36-mediated oxLDL uptake activates the NLRP3 inflammasome, resulting in the robust secretion of the inflammatory cytokine IL-1β36 [252]. In addition, oxLDL can bind to specific antibodies forming immune complexes that induce inflammatory responses in macrophages and dendritic cells. These immune complexes induce cellular activation, inflammatory cytokine production, and foam cell formation, mainly by signaling through the immune complex receptor Fc gamma receptor I (FcγRI) [253]. In addition, they can activate inflammasome via an FcγR/TLR4/CD36-dependent mechanism [254]. At early stages of atherosclerosis, the release of CCL2 and T-cell chemoattractants recruits monocytes and lymphocytes into the inner arterial wall, where monocytes are differentiated into macrophage foam cells under the influence of M-CSF. Upon fatty-streak lesion formation, T-cells secrete TNF-β, IFN-γ, fibrogenic mediators, and growth factors that induce the migration and proliferation of VSMCs. Activated T-cells also stimulate MMP production by macrophages in the lesion.

Cholesterol crystals activate NLRP3 inflammasome through lysosomal damage [255]. Several studies have shown that activated ECs overexpress cell adhesion molecules, such as VCAM-1, ICAM-1, PECAM-1, E-selectin, and P-selectin, on their surfaces [256]. Thus, these activated ECs are the local source of leukocyte recruitment into atherosclerotic lesions [257].

ECs, macrophages, and SMCs express LOX-1, which binds to oxLDL, leading to increased ROS production and VSMC death. This contributes to oxLDL infiltration into endothelium that disrupts the normal endothelial function, monocyte adhesion, and infiltration [258].

Prostaglandin (PG) E2 is an important lipid mediator that in a pathophysiological context mediates functions such as pyrexia, pain sensation, and inflammation. PGE2 is synthesized from arachidonic and by the participation of the cyclooxygenase (COX) enzyme and PGE2 synthase (PGES) afterward. It has been shown that a depletion of myeloid cell microsomal PGES, not in ECs or VSMCs, fosters atherogenesis in mice [259]. In addition, prostaglandin receptors (EP receptors) have been related to early atherosclerosis and the inflammatory process, which induce plaque erosion and rupture [260].

## 7. Post-Transcriptional Regulation of Atherosclerotic Plaques

Several studies have suggested that distorted non-coding RNA (ncRNA) expression and function are involved in the atherosclerotic process. Particularly, microRNAs (miRNAs) and long non-coding RNAs (lncRNAs) have been determined as important regulators in the progress of atherosclerotic plaque. While miRNAs are known to regulate gene expression post-transcriptionally, mainly through mRNA degradation, lncRNA-mediated regulation is less characterized, mainly due to its low sequence conservation and low expression. However, lncRNA can activate and repress genes by a variety of mechanisms at both transcriptional and translational levels, and its relevance in atherosclerosis development has been updated. It is now accepted that they play a crucial role in the atherosclerotic context [261,262].

### 7.1. miRNAs

miRNAs are crucial molecules for the maintenance of cardiovascular homeostasis [263]. Indeed, many studies in recent years have indicated that miRNA expression deregulation affects cellular and molecular processes that contribute to atherosclerosis. The pathological basis of atherosclerotic plaque formation and development is related to the expression levels of miRNAs (Table 1). Atherosclerotic coronary arteries revealed upregulated levels of miR-29, miR-100, miR-155, miR-199, miR-221, miR-363, miR-497, miR-508, and miR-181; on the contrary, miR-1273, miR-490, miR-24, and miR-1284 were found to be downregulated [264,265,266]. In the case of aorta, femoral, and carotid arteries, atherosclerotic plaques exhibited increased levels of miR-21, miR-34, miR-146, and miR-210 [267]. In addition, studies performed in carotid plaques showed an upregulation of miR-15, miR-26, miR-30, miR-98, miR-125, miR-152, miR-181, miR-100, miR-127, miR-133, miR-145, and miR-422 and a downregulation of miR-520 and miR-105 [268,269].

#### 7.1.1. miRNAs in Atherosclerotic Plaque Initiation and Progression

The first steps in atherosclerosis development include endothelial dysfunction, followed by inflammatory response and foam cell formation. There is an extensive list of miRNAs that regulate endothelial function, such as miR-221, miR-503, miR-217, miR-34a, miR-181b, miR-155, miR-126, miR-1, miR-223, miR-145, miR-146a, miR_92a, or miR10a (Table 2). Expression of miR-155 induces the downregulation of endothelin-1 and angiotensin II type I receptor, suggesting a role in endothelium protection [270]. Moreover, miR-10a showed an athero-protective role by repression of GATA6/VCAM1 signaling within ECs [271]. MiR-10a, miR-31, and miR-17-3p regulate inflammation modulating the expression of adhesion molecules in ECs [272]. MiR-126, one of the most studied miRNAs, plays a role in the prevention of atherosclerosis by regulating the vascular endothelial growth factor (VEGF) pathway and inhibiting endothelial permeability [273]. Another regulator of endothelial inflammatory response is miR-181b, which regulates the NF-kB pathway [274]. MiR-146a does not only decrease the lipid uptake in macrophages, suggesting an athero-protective role, but also inhibits endothelial activation by promoting eNOS expression [275]. MiR-125a reduces the lipid uptake, as well, through stimulated macrophages [276]. The expression of miR-223 negatively regulates cholesterol synthesis and exhibits an anti-inflammatory effect due to the attenuation of pro-inflammatory cytokine production [277]. On the contrary, miR-92a, a miRNA that reduces endothelial inflammation and atheroma plaque size, is upregulated in atheroma plaques via the regulation of Kruppel-like factor 2 (KLF2) [278,279]. Some miRNAs also modulate EC senescence. MiR-let-7g inhibits cellular senescence by regulating anti-aging gene sirtuin 1 (SIRT1) and the insulin growth factor (IGF) 1 pathway [280], while miR-126a was found to induce EC senescence via the activation of NF-kB signaling [281].

Furthermore, miRNAs also regulate the inflammatory response of macrophages during atherosclerosis development. These include, among others, miR-155, miR-222, miR-424, miR-503, miR-146a/b, and miR-147. MiR-125b overexpression induces the activation of macrophages by suppressing interferon regulatory factor 4 (IRF4) [282]. In addition, miR-342-p was found to enhance proinflammatory macrophage factors, such as IL-6. In animal models, it has been shown that the inhibition of miR-342-p reduces atheroma formation [283], while the inhibition of miR-33, a regulator of ABCA1 and ABCG1, reduces the cholesterol efflux to HDL in macrophages and promotes the development of atherosclerotic plaques in mice [284].

The regulation of VMSC proliferation is also regulated by miRNAs. MiR-21 overexpression induces the switch of VSMCs toward the synthetic phenotype, the one related to proliferation and ECM production [285]. Similarly, the inhibition of miR-221 and miR-222 diminishes the proliferative rate of VSMCs via the inhibition of c-Kit, p27 (Kip1), and p57 (Kip2) [286]. The synthetic VSMC phenotype is also induced by miR-26a through modulation of the TGF-β signaling pathway [287]. On the contrary, miR-143, miR-145, and miR-1 also play a role in preventing a VSMC phenotypic switch via the Kruppel-like factor 4 (KLF4), KLF5, and/or myocardin [288]. Myocardin also promotes a contractile phenotype via the inhibition of PDGF-β by miR-29a and miR-24 [289]. Similarly, miR-133a promotes the contractile phenotype in VSMCs, and its levels are decreased in atherosclerosis [288]. Moreover, the differentiation of VSMCs was also induced by miR-663, which promotes the expression of transcription factor JunB and myosin light chain 9 [290]. In addition, targeting the oncogene yes-associated protein (YAP) by miR-15b and miR-16 induced a more contractile phenotype in VSMCs [291]. Finally, several other miRNAs have been associated with a contractile and synthetic phenotype in VSMCs. Contractile-phenotype-associated miRNAs include miR195, miR-638, miR-7d, miR-100, miR-10a, miR-204, miR-199a, and miR-424; synthetic-phenotype-associated miRNAs include miR146a, miR-208, and miR-31. Here, we classified miRNAs related to a synthetic phenotype as anti-atherogenic (Table 2).

#### 7.1.2. miRNAs and Atherosclerotic Plaque Rupture

The mechanisms involved in plaque rupture are not completely understood, but plaque vulnerability is associated with fibrous cap thickness, necrotic core development, and the inflammatory response [293,294]. However, there is growing evidence that miRNAs play a role in processes leading to plaque rupture. MiR-322 is upregulated in vulnerable plaques compared with stable plaques, and its inhibition produces a downregulation of the proinflammatory cytokine IL-6 [295]. MiR-712 also leads to plaque rupture, as it activates a disintegrin and metalloproteinase with thrombospondin motifs 4 (ADAMST4) [296]. As mentioned before, the thickness of the fibrous cap depends on ECM synthesis and degradation, and it is associated with plaque vulnerability. The ECM is composed mainly of collagens, which are degraded by MMPs, and some miRNAs have been shown to target MMPs (i.e., miR-24 [265] and miR-133a [297]). MiR-494, which is expressed in atheroma plaques, downregulates TIMP3 and reduces the collagen content in the plaque [298]. Moreover, miR-29 helps to maintain the fibrous cap integrity targeting IFN-γ, which activates procollagen genes in VSMCs [299]. The inhibition of genes that produce ECM proteins by miR-29 may lead to plaque rupture [300]. Regarding inflammation in the necrotic core, miR-21 has been demonstrated to promote efferocytosis and suppress innate immune response [301]. In addition, miR-223, as a negative regulator of the NLRP3 inflammasome, prevents associated inflammatory response [302]. MiR-155 also contributes to the necrotic core formation in atherosclerosis, leading to vulnerable plaque through the induction of apoptosis [303]. In addition, miR-365 induces endothelial apoptosis, promoting plaque rupture [304]. Table 3 summarizes the role of these miRNAs in plaque rupture.

Although some miRNAs are considered signature molecules in atherosclerosis, more studies on miRNAs are needed to unravel the precise potential roles of miRNAs as targets for therapy.

#### 7.1.3. Distinct miRNA Regulation of VSMC and EC Function

Deciphering the role of different miRNAs in vascular biology as direct or indirect post-transcriptional regulators of VSMC and EC function constitutes a major challenge in the smooth muscle biology field. The discovery that the expression of miR-143/145 is critical for maintaining the contractile phenotype of VSMCs [305,306] highlighted the role of the bicistrionic unit encoding for miR-143 and miR-145 in the regulation of smooth muscle physiology [306,307]. The role of miR-143/145 was demonstrated in mice with a genetic deficiency of miR-143/145, which displays a reduced vascular tone and blood pressure control [308]. Additionally, the functional relevance of miR-143/145 in human vessel pathology has been suggested by the observed downregulation of the miR-143/145 cluster in human aortic aneurysms compared with normal aortic tissue [309].

Several studies have shown that cells can release miRNAs, which can then exert their specific effects by modulating processes in recipient cells. It has been shown that VSMCs communicate with ECs via miR-143/145 [310]. Cell-to-cell VSMC/EC contact induces the transcription of miR-143/145 in VSMCs, promoting the transfer of these miRNAs to the endothelium [310]. In particular, VSMCs can deliver miR-143/145 to ECs via membrane nanotubes or tunneling nanotubes [310]. The transfer of miR-143/145 from VSMCs to ECs is stimulated by TGF-β secreted by ECs. VSMC-derived miR-143/145 diminishes the angiogenic potential of the ECs by repressing hexokinase II and integrin β8 [310]. 

On the other hand, miR-126 acts as an intercellular messenger mainly released by ECs and internalized primarily by monocytes and VSMCs [311]. MiR-126 plays a critical role in modulating vascular development and homeostasis, targeting specific mRNAs, including the Sprouty-related protein 1 (SPRED-1), CXCL12, SDF-1, and phosphoinositol-3 kinase regulatory subunit 2 (PIK3R2) [312,313,314,315,316,317]. MiR-126 has also been related to the endothelial dysfunction associated with the development of diabetes and its complications [318].

### 7.2. lncRNAs

The progress achieved by next-generation sequencing technologies has revealed an increased number of lncRNAs associated with the pathogenesis of atherosclerosis [319]. Several lncRNAs identified in plaque tissues play a protective role in vascular disease. lncRNA *MeXis* (Macrophage-expressed LXR-induced sequence) is involved in cholesterol transport. Animal models lacking *MeXis* showed an increased atherosclerotic load and a decreased expression of the atheroprotective protein ABCA1 in atheroma plaques [320]. Reduced expression of lncRNA *MALAT1* (metastasis-associated lung adenocarcinoma transcript 1) was also found to be associated with the development of atheroma plaques [321]. The knockdown of *MALAT1* promotes lipid uptake in foam cells by inducing the transcription of scavenger receptor CD36 [322]. Moreover, lncRNA *CHROME* (cholesterol homeostasis regulator of miRNA expression) protects against atherosclerosis promoting cholesterol efflux by inhibiting miRNAs, such as miR-33 [323,324]. However, few lncRNA have shown athero-protective effects by inhibiting apoptosis and senescence. In particular, lncRNA *CERNA1* promotes plaque stabilization by inhibiting cellular apoptosis via inducing API5 expression [325], and lncRNA *SNHG12* in pig and human atheroma plaques inhibits DNA damage and senescence [326]. Human carotid atherosclerotic plaques show decreased levels of lncRNA *NEXN-AS1* and lncRNA *MANTIS* [327,328]. *NEXN-AS1* upregulates the expression of *NEXN*, a gene that exerts atheroprotective effects in VSMCs and ECs [327]. Furthermore, one lncRNA, called *SENCR* (smooth muscle and EC-enriched migration/differentiation-associated long noncoding RNA), plays a role in atherosclerosis development by protecting the endothelial layer [329]. Hence, Boulberda et al. [330] found altered levels of lncRNA *SENCR* in vascular tissue. Moreover, the newly identified lncRNA *RP11-714G18.1* is also downregulated in atheroma plaques. This lncRNA inhibits VSMC and EC migration by downregulating the expression of MMP1, inhibiting atherosclerosis progression. In addition, *RP11-714G18.1* inhibits the adhesion of monocytes to ECs [331].

A recent study highlighted the role of lncRNA *SMILR* in atherosclerosis and showed that its expression is significantly higher in unstable plaques than in stable plaques and increases VSMC proliferation by regulating the expression of the proximal gene HAS2 [332]. Moreover, lncRNA *SMILR* binds directly to the mRNA of the mitotic protein CENPF (centromere protein F) driving the proliferation of smooth muscle cells [332]. The aberrant proliferation and migration of VSMCs are critical factors in atherosclerotic plaque formation, and several molecular mechanisms that involve lncRNAs control these processes. Among the lncRNAs that suppress VSMC migration, lncRNA *RP11-714G18.1* acts by directly targeting LDL-related receptor 2 binding protein (LRP2BP) in atherosclerosis [331]. Very recently, it has been found that lncRNA *ZNF800*, which is highly expressed in human atherosclerotic plaque tissues and predominantly in VSMCs, suppresses VSMC proliferation and migration through the AKT/mTOR/HIF-1α signaling pathway by activating PTEN [333]. Another study demonstrated that lncRNA *RNCR3* expression is significantly higher in atherosclerotic lesions, leading to reduced VSMC proliferation and migration via the *RNCR3*/Kruppel-like factor 2/miR-185-5p regulatory network [334]. Furthermore, lncRNA *RP11-714G18.1* impairs VSMC migration in atherosclerosis through the LRP2BP-mediated downregulation of MMP1 [331]. The expression of lncRNA *p21* is also reduced in atherosclerotic plaques, and it represses VSMC proliferation and atherosclerosis by enhancing TP53 activity, thus playing an atheroprotective role in atherosclerosis [335]. 

Similarly, lncRNA *CCL2* is upregulated in unstable atherosclerotic plaques compared to stable atherosclerotic plaques. Moreover, lncRNA *CCL2* modifies the mRNA levels of the pro-inflammatory chemokine CCL2 (or MCP-1) by interacting with RNA binding proteins (HNRNPU and IGF2BP2) [336]. Recently, lncRNA *GAS5* (growth arrest-specific 5) has received attention as a potential biomarker for atherosclerosis [337]. Levels of *GAS5* were upregulated in atherosclerotic plaques, and *GAS5* binds and suppresses the microRNA miR-221, increasing the production of MMPs and pro-inflammatory molecules within the atherosclerotic plaque [338]. Arslan et al. [321] have identified the upregulation of lncRNA *MIAT* (myocardial infarction-associated transcript) in atheroma plaques. *MIAT* increases VSMC proliferation by binding and suppressing miR-181b [339]. Another lncRNA that displayed effects on VSMC proliferation and migration is *BANCR* (*BRAF*-regulated lncRNA 1) [340]. Finally, lncRNA *ANRIL* (antisense non-coding RNA in the *INK4* locus) is also a crucial molecule in atherogenesis, as it affects several cell types in the atherosclerotic plaque, where it is upregulated and is directly correlated with atherosclerosis severity [341,342]. Recently, circular forms of *ANRIL* (*circANRIL*), comprising different exons and opposed to the linear form, have been identified. Conversely, *circANRIL* is inversely correlated with atherosclerosis risk [343]. *CircANRIL* has been found in vascular tissue, smooth muscle cells, and macrophages, where it exhibits an athero-protection function [342].

In summary, recent findings suggest that lncRNA plays a crucial role in the development of atherosclerotic plaque (Table 4). However, for most of them, the specific mode of action is not completely understood, and further research is needed to understand the complex role of lncRNA in atherosclerosis.

## 8. Microbiota

The human body hosts many bacteria species (microbiota) that have co-evolved with humans, and they have generated a symbiotic relationship that mutually benefit each other. Microbiota is able to positively affect human physiology through the synthesis of different metabolites or the training of the immune system; however, it has also been associated with the development of different diseases, such as atherosclerosis or cardiovascular disease (CVD) [344,345].

The presence of microorganisms in the atheroma plaque is well established, and some studies have shown that the abundance of certain phyla could correlate with plaque stability and overall inflammation [346,347]. For instance, bacterial DNA from pathogenic families, such as *Helicobacteraceae* or *Neisseriaceae*, has been shown to be more abundant in plaques of symptomatic patients [348,349]. Nevertheless, these results need to be corroborated in larger studies.

Gut microbiota also contributes to the development of atherosclerosis and CVD. The gut hosts the largest microbial population in humans and is able to regulate many biological functions, such as energy storage, the absorption and processing of nutrients, or the maturation of the immune system [350,351]. However, when gut microbial homeostasis is disrupted (referred to as dysbiosis) toward underrepresented colonies, it can induce the development of different diseases, such as cancer, type 2 diabetes, obesity, CVD, and atherosclerosis [346,352,353]. In addition, gut dysbiosis increases intestinal permeability, reducing the expression of tight junction proteins and allowing the translocation of lipopolysaccharides that induce low-grade inflammation via Toll-like receptors [354]. On the other hand, several metabolites produced by the gut microbiota can modulate systemic inflammation and contribute or prevent atherosclerosis development. The most studied one is trimethylamine-N-oxide (TMAO) [355], the oxidized form of trimethylamine (TMA). TMA is synthesized by gut microbiota after metabolizing diet choline and carnitine. It is then transferred to the bloodstream and oxidized to TMAO in the liver through flavin mono-oxigenase [356]. TMAO increases vascular wall inflammatory responses and suppresses reverse cholesterol transport favoring cholesterol accumulation in the intima [357]. Moreover, APOE^−/−^ mice fed with a choline-rich diet showed increased TMAO levels and atheroma plaque development, a process that was reversed by antibiotic intervention [358].

Indirect infections in distant areas from atheroma plaques also contribute to its development and destabilization. Indeed, in recent years, many studies have correlated the incidence of atherosclerotic cardiovascular disease (ACV) with bacterial infections. Epidemiological studies support the idea of a causative association between periodontal disease or *chlamydia* infections and CVD [359]. A large study that included almost 12,000 participants demonstrated that poor oral hygiene is associated with an increased rate of CVD and increased lower-grade inflammation [360]. Moreover, a small study with 92 participants showed that some bacteria phyla were more abundant in the oral cavity of patients with symptomatic atherosclerosis than in control patients, indicating a possible association between oral bacteria and atheroma plaque development [361]. Nevertheless, the direct association of bacteria in the oral cavity and ACV is still controversial [362].

## 9. Sex as an Important Risk Factor in Atherosclerosis

Several modifiable and non-modifiable atherosclerotic risk factors have been well described. Among them, unhealthy diet, physical inactivity, dyslipidemia, hyperglycemia, high blood pressure, obesity, sex, and age [363,364,365,366]. Here, the impact of sex on atherosclerosis will be covered in detail.

The pathophysiology of atherosclerosis shows different patterns between women and men due to inherent biological and social differences (e.g., Younis, N.). In 1948, the US Public Health Service initiated the Framingham study, which indicated that sexual dimorphism in major modifiable risk factors, including cigarette smoking, dyslipidemia, hypertension, and diabetes mellitus, could be responsible for the observed differences in atherosclerosis development and/or complications [367]. Since then, large datasets have shown that the impact of these risk factors on the associated risk of myocardial infarction (MI) is higher in women compared to men, with odds ratios of 1.3, 1.5, and 1.6, for smoking, hypertension, and diabetes mellitus, respectively [368,369]. In addition, modern epidemiological data show that younger women have a decreased risk to develop CVD and lower rates of MI relative to men [370,371]. However, the observed cardioprotective effect in women is lost between the age of 60 and 79 years, and CVD risk in women surpasses that in men by the age of 80 years [371]. Nonetheless, this pattern of disease onset is not recapitulated in stroke, which shows a greater prevalence in women until the seventh decade of life and a greater probability of recurrent stroke within the first 5 years after stroke compared to men [372]. Additionally, coronary artery disease (CAD) is often underdiagnosed and viewed as a leading cause of female mortality. In Europe, CVDs account for 43% of deaths in men and 55% in women [373]. When analyzing the different components of CVDs, coronary heart disease (CHD) represents 21% of deaths in men and 23% in women, whereas, as indicated above, stroke is a more frequent cause of death in women than in men (18 and 11%, respectively), as well as the other CVDs (15% in women and 11% in men). These demographic statistics evidence sex differences in CVD risk and highlight the need to take into consideration sex as an important variable to be included from animal models through clinical trials [372].

**Figure 4:**
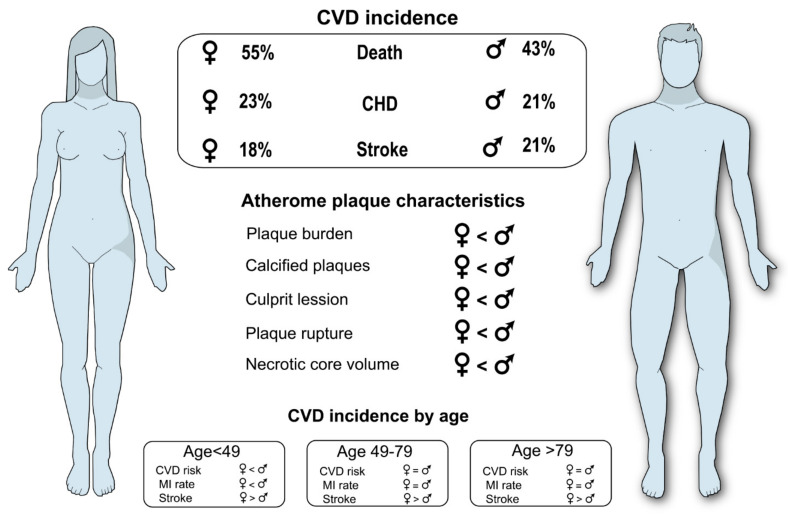
Different patterns in CVD incidence between women and men.

Although great efforts have been made to understand molecular mechanisms and discover novel drug targets for atherosclerosis, data examining sex differences are still relatively limited. A robust study, in which 771 preclinical articles on atherosclerosis and other vascular diseases were analyzed, showed that the sex of animals is not reported in the 18.8% of them. When the sex was specified, 55.4% of the studies were performed on males, 20.4% were performed on females, and less than 25% studied both males and females [374]. The proportion of studies including both sexes was very similar (21–28%) in recently published articles [375,376]. Moreover, fewer than half of the studies including both sexes directly compare males and females with the appropriate statistical analysis to consider sex as an independent variable or the interaction of sex with a treatment or genotype [374,375,376]. Different patterns in CVD incidence between women and men are shown in Figure 4.

### 9.1. Impact of Plaque Size and Morphology between Sexes

Frequently, exertional ischemia (i.e., angina and claudication) occurs when an individual plaque enlarges sufficiently to impair blood flow to meet tissue demand (usually >70% stenosis). On the other hand, plaque rupture leads most of the morbidity and mortality events from atherosclerosis, such as MI, stroke, disabling peripheral artery disease, and eventually death. Non-invasive imaging has shown that men develop plaques earlier and have a greater plaque burden than women, even after accounting for differences in risk factors. In addition, post-mortem pathological studies have shown that plaques with 30–40% stenosis are more likely to rupture, leading to vascular occlusion and death [377]. These studies suggest that an overall plaque burden, a plaque inflammatory state, and an unstable plaque morphology contribute to acute MI and stroke risk, in agreement with the greater incidence of ischemic events in males, although this relationship changes later in time [378,379,380]. Additionally, it has been shown that women do not present as much of an atherosclerotic plaque burden as men and have fewer high-risk plaque features [381,382,383]. Significantly more atherosclerotic and calcified plaques, higher rates of CAD, and major adverse cardiac events over 5.6 years of follow-up have been observed in men compared with women [380]. Moreover, the cross-sectional REFINE-Reykjavik study, which included 21,132 patients, showed that 50% of women had normal computed tomography scans compared with 31% of men [378].

New imaging techniques allow one to assess individual atherosclerotic plaque features that are associated with adverse events. Intravascular ultrasound, which provides information about both the degree of stenosis and the extent of necrotic core in the plaque, has shown that women suffering from acute coronary syndrome present a similar number of culprits compared to men [379]. Nevertheless, despite a higher mean age and multiple comorbidities, women present fewer non-culprit lesions, fewer involved coronary arteries with lesions, a lower frequency of plaque rupture, and a smaller total necrotic core volume [381,384].

### 9.2. Clinical Implications

The importance of understanding age and gender differences in culprit plaque composition is crucial to choose more appropriate pharmacological and interventional therapies [385]. The significantly higher prevalence of thin-cap fibroatheroma in elderly women patients compared to men shows the importance of intensive lipid-lowering therapy for women [386]. However, recent studies found that women were less likely to be eligible for statin administration than men, due to women being offered statins at a lower rate by their caregivers [387,388,389]. Sufficient intensity and duration of lipid-lowering therapy, including statins, should be provided for women as that provided for men [386]. With the imbalance of cholesterol metabolism being a key factor in the progression of atherosclerosis by increasing age in women, ezetimibe in combination with statins might also be effective to stabilize coronary plaque in women [390,391].

In contrast to the treatment and prevention of vulnerable plaque, the therapeutic strategy for coronary calcification may be more complex and challenging. For the interventional treatment of culprit lesions with large calcification, intracoronary imaging techniques may provide safer procedures and better clinical outcomes [392,393]. Nonetheless, lesions with large plaques also require the use of a debulking device, and a subsequent wider stent area is also required for calcification [394]. In addition, lifestyle modification may be more important in women than in men regarding the risk reduction of atherosclerotic change in plaque components and subsequent disease onset [395,396], as they are considered modifiable factors.

## 10. Cigarette-Smoking-Induced Atherosclerosis

Cigarette smoking is a powerful independent risk factor for atherosclerosis and ACV events, since chemical constituents of smoke have high oxidant and inflammatory capacities that can directly induce endothelial damage and potentiate inflammatory response [397,398,399,400,401]. Clinical evidence has shown a direct proportional dose-dependent association of smoking exposure with the presence of extensive and calcified atherosclerotic plaques, and smoking cessation at any age is one of the most important health interventions for reducing risks of ACV, cancer, and mortality [402]. Smoking cessation has been associated with less progression of carotid plaque, but not with intima media-thickness [403]. The effects of smoking cessation on carotid atherosclerosis were related to the degree of abstinence and persisted after adjusting for baseline smoking heaviness and ACV risk factors [403]. 

### Molecular Mechanisms Underlying Clinical Smoking-Induced Atherosclerosis

It has been shown that single smoking compounds (e.g., nicotine, carbonyl compounds, acrolein, and methyl vinyl ketone) and/or their combined action can affect each stage of the atherosclerotic process [401]. 

Exposition to smoke can promote oxidative stress, which constitutes one of the main mechanisms underlying endothelial injury. As mentioned above, oxidative stress influences the activity of numerous enzymes (e.g., eNOS and NADPH–oxidases) [404] and leads to irreversible modification of different proteins, thereby deeply altering intracellular signaling pathways. Smoke is able to increase LDL levels through metabolic alterations and the induction of LDL oxidation due to the direct oxidant capacity of smoke components [401].

Smoke exposition also leads to NF-κB activation in ECs, increasing the expression of adhesion molecules on the plasma membrane [405] and the upregulation of inflammatory genes, including IL-1 and COX-2 [406]. Furthermore, nicotine triggers the secretion of pro-inflammatory adipokines from the perivascular adipose tissue [407]. 

Moreover, smoking induces the proliferation and migration of VSMCs and the switch from contractile to secretory phenotype by increasing the expression of IFN-β and PDGF [305,408]. Consequently, VSMCs are able to release pro-inflammatory factors and extracellular matrix components. 

Atherosclerotic plaques of smokers are characterized by a predominance of the lipid core, and the fibrotic cap is thinner than in non-smokers [409]. This configuration is partly due to augmented MMP activity in the plaque of smokers [410,411]. Smoking also triggers the infiltration and activation of macrophages inside the lesion, as well as their conversion in foam cells, contributing to the growth of the lipid core. Smoking is also essential in the process of platelet activation and adhesion to endothelium [412]. In addition to these effects, smoking can cause an increase in blood pressure, a determinant for plaque damage and instability. 

Several microRNA pathways that are involved in the development of atherosclerosis are also regulated by cigarette-smoke exposure [413,414]. It has been shown that exposure to high-dose cigarette smoke leads to the upregulation of miRNA-155 and miRNA-21, which target PPAR-α. The downregulation of PPAR-α leads to the upregulation of VCAM-1, ICAM-1, and MCP1 through the activation of transcription factor AP-1 [415,416]. VCAM-1 and ICAM-1 mediate the firm adhesion of leukocytes to the ECs and play a critical role in subsequent leukocyte transmigration, leading to atherosclerosis [417,418], whereas MCP-1 regulates the migration and infiltration of monocytes/macrophages [419]. 

In addition, the upregulation of miR-155 and miR-21 modulates the EGFR/ERK/p38 MAPK and the PI3K/Akt/eNOS pathways in ECs, respectively [420,421]. 

## 11. Conclusions

During the last few years, we are witnessing an increased burden of atherosclerotic disease that contributes to CVD risk, which is becoming a global epidemic. The study of cellular and molecular biology mechanisms of atherosclerosis has provided remarkable insights into the processes that lead to atheroma development and the clinical manifestations of this disease. Knowledge and continued research about the functions of non-coding RNAs in plaque development have improved, showing that miRNAs and lncRNAs alter the transcription of genes implicated in atherosclerosis. In addition, microbiota have been linked to the development of atherosclerosis by identifying microbial ecosystems residing in different habitats of the human body that contribute to metabolic and cardiovascular disorders. The role of microbiota in atherosclerosis development is supported by increasing mechanistic evidence; however, further studies are needed to understand the contribution of microbiota to atherosclerosis. Finally, the importance of analyzing sex-specific differences as risk factors associated with atherosclerosis is important for individualized risk-management strategies to prevent the development and progression of atherosclerosis.

The progress in understanding the mechanisms that lead to atherosclerosis development will surely provide therapies to address the unacceptable burden of persistent risk and will ultimately improve the diagnosis and prognosis of the disease.

## Figures and Tables

**Table 1:** Differential microRNA expression levels in atherosclerotic arteries.

Location	Upregulated	Downregulated
Coronary arteries	miR-29, miR-100, miR-155, miR-199, miR-221, miR-363, miR-497, miR-508 and miR-181 [264,265,266].	miR-1273, miR-490, miR-24 and miR-1284 [264,265,266].
Aorta, femoral, and carotid arteries	miR-21, miR-34, miR-146 and miR-210 [267]. Only in carotid plaques: miR-15, miR-26, miR-30, miR-98, miR-125, miR-152, miR-181, miR-100, miR-127, miR-133, miR-145 and miR-422 [268,269].	Only in carotid plaques: miR-520, miR-105 [268,269].

**Table 2:** Role of microRNAs in atherosclerosis initiation and progression.

Cell Line	Athero-Protective	Pro-Atherogenic
Endothelial cells	miR-155 [270] miR-10a [271], miR-31 and miR-17-3p [272] miR-146a [275] miR-181b [274] miR-92a [278,279] miR-let-7g [280]	miR-216a [281]
Macrophages	miR-146a [275] miR-125a [276] miR-223 [277]	miR-125b [282] miR-342-p [283] miR-33 [292]
VSMCs *	miR-21 [285] miR-221 and miR-222 [286] miR-26a [287] miR-663 [290]	miR-143, miR-145 and miR-1 [288] miR-29a and miR-24 [289] miR-133a [288] miR-15b and miR-16 [291]

* Anti-atherogenic, considered as VSMC activation and phenotype switching.

**Table 3:** Role of microRNA in plaque rupture.

Anti-Atherogenic	Pro-Atherogenic
miR-24 [265]	miR-322 [295]
miR-133a [297]	miR-712 [296]
miR-29 [299,300]	miR-494 [298]
miR-21 [301]	miR-155 [303]
miR-223 [302]	miR-365 [304]

**Table 4:** Role of lncRNA in atherosclerosis.

Athero-Protective	Pro-Atherogenic
*MeXis* [304] *MALAT1* [321,322] *CERNA1* [325] *SNHG12* [326] *NEXN-AS1* [327] *MANTIS* [328] *SENCR* [329] *RP11-714G18.1* [331] *CHROME* [323,324] *circANRIL* [342]	*SMILR* [332] *CCL2* [336] *GAS5* [337,338] *MIAT* [321,339] *BANCR* [340] *ANRIL* [341,342]
